# Antiviral effect of melatonin on Japanese encephalitis virus infection involves inhibition of neuronal apoptosis and neuroinflammation in SH-SY5Y cells

**DOI:** 10.1038/s41598-023-33254-4

**Published:** 2023-04-13

**Authors:** Kuntida Kitidee, Arisara Samutpong, Nattaporn Pakpian, Tanchanok Wisitponchai, Piyarat Govitrapong, Russel J. Reiter, Prapimpun Wongchitrat

**Affiliations:** 1 Center for Research Innovation and Biomedical Informatics, Faculty of Medical Technology, Mahidol University, 999 Phutthamonthon 4 Road, Salaya, Nakhon Pathom, 73170 Thailand; 2 Department of Biomedical Engineering, School of Engineering, King Mongkut’s Institute of Technology Ladkrabang, Bangkok, Thailand; 3 Chulabhorn Graduate Institute, Chulabhorn Royal Academy, Bangkok, Thailand; 4 Department of Cell Systems and Anatomy, UT Health San Antonio, San Antonio, TX USA

**Keywords:** Antivirals, Cell death in the nervous system

## Abstract

Japanese encephalitis virus (JEV), a mosquito-borne flavivirus, causes high mortality rates in humans and it is the most clinically important and common cause of viral encephalitis in Asia. To date, there is no specific treatment for JEV infection. Melatonin, a neurotropic hormone, is reported to be effective in combating various bacterial and viral infections. However, the effects of melatonin on JEV infection have not yet been studied. The investigation tested the antiviral effects of melatonin against JEV infection and elucidated the possible molecular mechanisms of inhibition. Melatonin inhibited the viral production in JEV-infected SH-SY5Y cells in a time- and dose-dependent manner. Time-of-addition assays demonstrated a potent inhibitory effect of melatonin at the post-entry stage of viral replication. Molecular docking analysis revealed that melatonin negatively affected viral replication by interfering with physiological function and/or enzymatic activity of both JEV nonstructural 3 (NS3) and NS5 protein, suggesting a possible underlying mechanism of JEV replication inhibition. Moreover, treatment with melatonin reduced neuronal apoptosis and inhibited neuroinflammation induced by JEV infection. The present findings reveal a new property of melatonin as a potential molecule for the further development of anti-JEV agents and treatment of JEV infection.

## Introduction

Japanese encephalitis is the most commonly diagnosed epidemic encephalitis in the world and is among the most significant viral encephalitides in Asia. Japanese encephalitis is caused by an infection with the Japanese encephalitis virus (JEV), a mosquito-borne flavivirus. Infection by JEV can cause acute encephalitis with a high mortality rate in humans. Over 3 billion people in 25 countries in Asia, along with northern Australia and some parts of the western Pacific, are at risk of JEV infection^1^. The most comprehensive estimate of incidence within the past decade suggests that an estimated 70,000 cases of Japanese encephalitis occur every year. Approximately 25% of Japanese encephalitis patients die, while 30–50% of survivors will experience permanent neurological or psychiatric sequelae, including memory loss, impaired cognition, language impairments, behavioral disturbances, convulsions, motor weakness or paralysis, and abnormalities in tone and coordination^2,3^. The subsequent permanent neuronal abnormalities negatively affect the quality of life in both patients and their families.

JEV is a member of the genus *Flavivirus* in the family *Flaviviridae*, which includes dengue (DENV), West Nile (WNV) and Zika viruses (ZIKV). JEV is an arbovirus maintained in a zoonotic cycle. The main JEV transmission cycle involves *Culex tritaeniorhynchus* mosquitoes and similar species that lay eggs in rice paddies and other open water resources, with pigs and aquatic birds as principal vertebrate amplifying hosts with humans generally being considered dead-end JEV hosts^4^. JEV particles are small in size (~ 50 nm), and they consist of a glycoprotein-containing lipid envelope surrounding the capsid, which encases a single-stranded positive-sense 11-kb RNA genome. The viral RNA carries a single open reading frame with genes for three structural proteins, i.e., capsid (C), premembrane (prM), and envelope (E), and seven nonstructural (NS) proteins, i.e., NS1, NS2a, NS2b, NS3, NS4a, NS4b, and NS5^3^.

The invasion of the central nervous system (CNS) by the JEV is associated with neurodegeneration due to oxidative stress, leading to neuronal cell death^5–7^. Additionally, JEV infection induces a rapid inflammatory response with inflammatory cell infiltration into the brain parenchyma and phagocytosis of the infected cells^8^. Microglial activation and subsequent release of various proinflammatory mediators induce neuronal death following JEV infection^9^. Although vaccines for Japanese encephalitis are available, the disease is difficult to eradicate owing to its zoonotic cycle and the incomplete effectiveness of the vaccine used^10^. To date, no specific drugs are available to treat Japanese encephalitis. Hence, intensive care, treatment and support are still needed to lower the death rate. It is essential that any effective therapy must relieve symptoms and stabilize patients. Therefore, finding effective antiviral agents against JEV infection is necessary.

**Figure 1:**
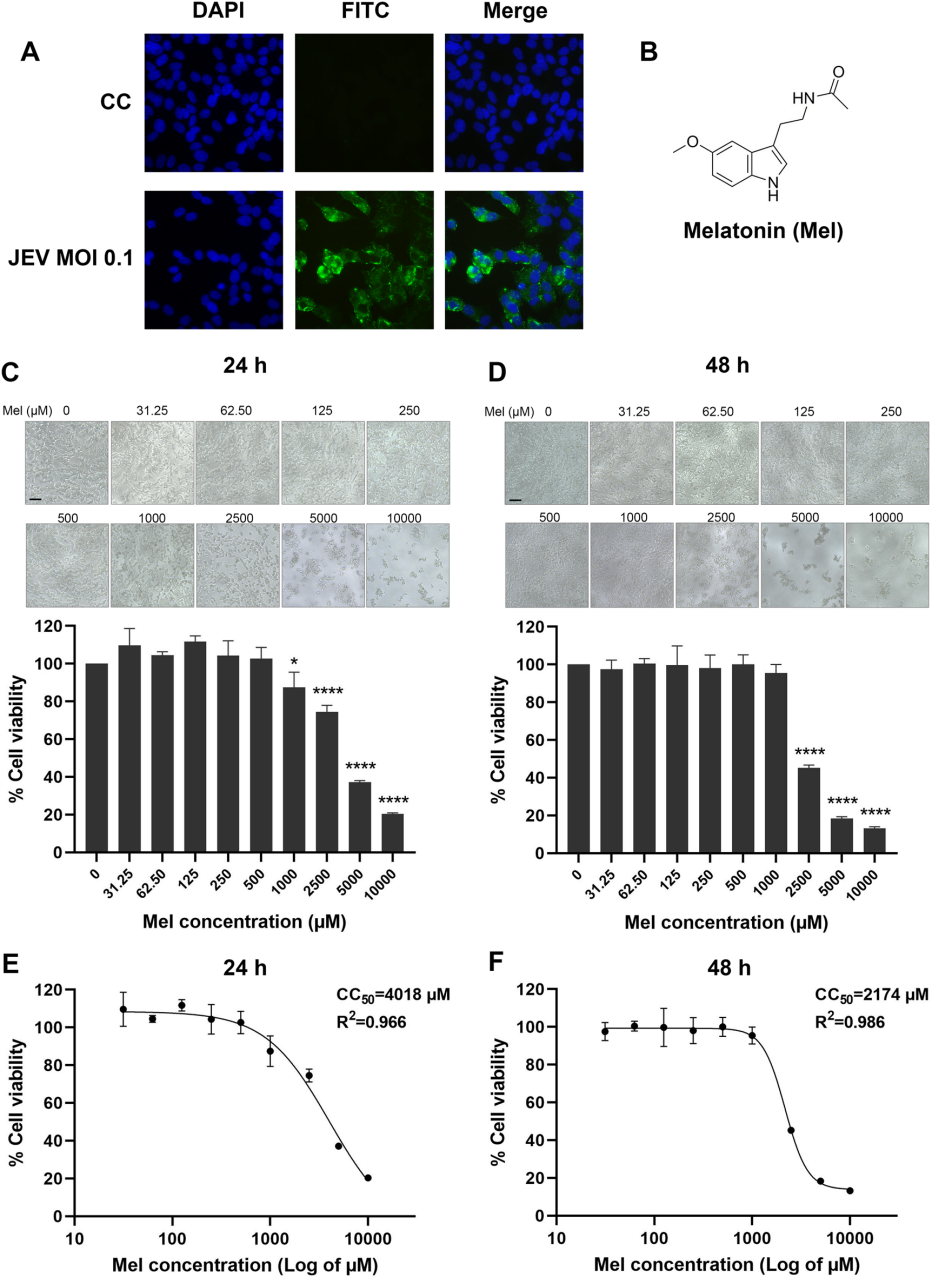
(**A**) Immunofluorescence analysis of viral envelope protein (green) expression in Japanese encephalitis virus (JEV)-infected SH-SY5Y cells. (**B**) Chemical structure of melatonin (Mel). (**C**) Viability of SH-SY5Y cells at 24 h. (**D**) Viability of SH-SY5Y cells at 48 h. The cells were treated with melatonin at various concentrations, and the cell viability was determined at 24 h using the MTS assay. The percentage of cell viability is presented compared to the cell control. The graph shows the means ± SD of three independent experiments. Representative image of the cell viability assay was presented above (magnification, × 40). Scale bar are 10 µm. The CC_50_ values of melatonin for SH-SY5Y at (**E**) 24 h and (**F**) 48 h was calculated using GraphPad Prism by dose–response-inhibition equation. One-way ANOVA and Tukey–Kramer multiple comparisons tests were performed for statistical analysis. *p < 0.05 and ****p < 0.0001, compared to the control without melatonin treatment (0 μM).

Melatonin (N-acetyl-5-methoxytryptamine, Fig. 1B) is a neurohormone produced in and secreted by the pineal gland. Melatonin is amphiphilic, and readily enters cells where it functions intracellularly in a receptor-independent manner; melatonin also has action via specific receptors^11–13^. Melatonin plays a fundamental role in promoting the neuroimmune-endocrine system but is also function as a potent antioxidant with therapeutic potential in numerous diseases. For example, melatonin is effective in preventing cell damage under acute (sepsis, asphyxia in newborns) and chronic conditions (metabolic and neurodegenerative diseases, cancer, inflammation, aging)^14^. In addition, melatonin has also been found to be effective in combating various bacteria, parasites, fungi and viruses^15,16^. Melatonin supplementation has been shown to protect against several neurotrophic viral infections, including WNV-25, Semliki Forest virus (SFV), encephalomyocarditis virus (EMCV), and Venezuelan equine encephalomyelitis virus (VEEV)^17,18^. However, the ability of melatonin to modulate the JEV infection has not been investigated. Supporting the use of melatonin for this purpose, animal models and in vitro experiments have revealed that a number of antioxidants and immune-inflammatory modulators, actions that melatonin possesses, are potentially effective for the treatment of JEV infection^6,10^. With this in mind, the present study aims to determine (1) the antiviral activity of melatonin against JEV infection in vitro by focusing on the effect on JEV yields in neuronal cells, and tested (2) the neuroprotective effect of melatonin against JEV-induced neuronal cell death via apoptosis pathways and molecular mechanisms of neuroinflammation.

## Materials and methods

### Virus and cell lines

The Beijing strain of JEV was kindly provided by Prof. Dr. Sutee Yoksan at the Center for Vaccine Development, Institute of Molecular Biosciences, Mahidol University. The identity of the virus stock was confirmed and propagated in Vero cells (African green monkey kidney cells, ATCC No. CCL-81) for subsequent use as described in our previous study^5^. Virus stocks were quantified by plaque assay and stored at − 80 °C until use. SH-SY5Y cells (human neuroblastoma, ATCC No. CRL-2266) were cultured in Dulbecco's modified Eagle's medium (DMEM) containing 10% heat-inactivated fetal bovine serum (FBS) at 37 °C and 5% CO_2_ until a sufficient number of cells was obtained for experiments.

### Cytotoxicity of melatonin

The analytical grade melatonin was purchased from Sigma-Aldrich (St. Louis, MO, USA). Melatonin solution was freshly prepared by first dissolving in a small volume of 40% ethanol before being diluted in 2% FBS-supplemented medium to the indicated concentration. Melatonin was protected from light during usage. For melatonin treatment, a confluent monolayer of SH-SY5Y cell lines was prepared in 96-well microplates and treated with various concentrations of melatonin (31.25, 62.50, 125, 250, 500, 1000, 2500, 5000 and 10,000 μM) for 24 and 48 h. Assays were run in quadruplicates and 2% FBS-supplemented medium was used as a control. After treatments, cell viability was evaluated by MTS assays according to the manufacturer’s protocol of CellTiter 96^®^ AQueous One Solution Cell Proliferation (Promega, Madison, WI, USA). The optical densities were measured by a Synergy HTX Multi-Mode Reader (BioTek, Winooski, VT, USA) at 490 nm. Cell viabilities were calculated as a percentage of the total number of viable control cells. The 50% cytotoxic concentration (CC_50_) of melatonin was defined as the concentration that reduced half of the cells, was calculated. The concentration of melatonin with the least cytotoxicity and below CC_50_ value was selected and used for subsequent experiments.

### Virucidal assay

The extracellular effects of melatonin against JEV particles were investigated by incubating the JEV suspension at 10^5^ plaque forming units (pfu) with an equal volume of medium containing various concentrations of melatonin (250, 500, 1000, 2500 and 5000 μM) for 2 h at 37 °C and 5% CO_2_. Medium containing 2% FBS was used as a control. After incubation, the treated viral suspension was used to determine the remaining viral titer by performing the plaque assay.

### Pre- and post-treatment assay

SH-SY5Y cells were seeded in 24-well plates until they reached 80–90% confluence in 24 h and then pretreated with melatonin (125, 250 and 500 μM) for 1 h. After melatonin was removed, the cells were infected with JEV (MOI 0.1) for 1 h. Subsequently, the infection mixtures were replaced with culture medium containing same concentrations of melatonin as pre-treatment for the rest of the treatment period. A schematic of viral infection and melatonin treatment is shown in Fig. 3A. The supernatant was collected 24 and 48 h post infection (hpi) to determine the change in virus titer using the plaque assay. The percent inhibition was evaluated at 24 and 48 hpi.

### Time-of-addition assay

The antiviral mechanism of melatonin was investigated by a time-of-addition assay. SH-SY5Y cells were seeded in 24-well plates until they reached 80–90% confluence in 24 h and then were infected with JEV (MOI 0.1). Melatonin at selected concentrations (125, 250 and 500 μM) was added at specified time points. A schematic of the virus infection and melatonin treatment regimens is shown in Fig. 4A. Cells were subjected to following treatment regimens: (1) pre-infection, cells were pretreated with melatonin for 1 h prior to virus adsorption; (2) during infection, cells were infected with JEV in the presence of melatonin for 1 h. Infection mixtures were subsequently removed. (3) post-infection, melatonin was added after virus inoculation for the rest of the experiments. Cells were infected with JEV and left untreated as a control of infection. The supernatant from each treatment group was collected 24 and 48 hpi. The reduction in virus titer was determined by the plaque assay in Vero cells. The percentage of inhibition in each condition was evaluated at 24 and 48 hpi.

### Determination of viral titer using the plaque forming assay

The viral titer was measured by the plaque assay as described in our previous report^5^. Briefly, Vero cell monolayers were prepared in 12-well culture plates and infected with tenfold diluted JEV suspension at 37 °C for 1 h. Control cells were incubated with viral growth media. After removing the inoculum, the cells were overlaid with 1.5% carboxymethylcellulose containing EMEM with 2% FBS for 6 days at 37 °C. Cells were fixed with 10% formaldehyde for 1 h followed by staining with crystal violet for 15 min. JEV plaques were averaged from triplicate wells and presented as pfu/mL.

### Molecular docking analysis

To investigate the possible inhibitory activities of melatonin against JEV replication, the JEV replicational enzymes NS3 and NS5 were selected for molecular docking analysis. The NS3 protein consists of an N-terminal protease domain (NS3Pro) and a C-terminal RNA helicase domain (NS3Hel). NS5 harbors an N-terminal RNA methyltransferase (NS5MTase) domain and a C-terminal RNA-dependent RNA polymerase (NS5RdRp) domain. The sequence of the JEV polyprotein (strain Beijing-1; GenBank No. L48961.1) was retrieved from the NCBI database [accessed 2020 Jan 15], and it was used to search for crystal structures of JEV NS3 and NS5 in the Protein Data Bank through SWISS-MODEL (last update 25 July 2020)^19^. The crystal structures of JEV NS3Pro (PDB ID: 4R8T^20^), JEV NS3Hel (PDB ID: 2Z83^21^), JEV NS5MTase (PDB ID: 4K6M^22^), and NS5RdRp (PDB ID: 4K6M^22^) that shared 98.58%, 99.54%, 100%, and 99.03% sequence identity with the Beijing-1 strain were obtained from the PDB. An individual protein of each PDB entry was used to add hydrogen atoms, calculate Kollman charges, and generate PDBQT files using AutoDockTools^23^.

Meanwhile, the melatonin moiety was extracted from PDB ID: 5MXW^24^ and docking simulation was performed as described above without adding the Gasteiger charges. During blind docking simulation in the absence of water molecules, melatonin was treated as flexible, and the rotatable torsions of melatonin were defined using AutoDockTools. The 1000 docked poses of melatonin with NS3Pro or NS5MTase were generated in the grid-box dimension of 140 × 140 × 140, whereas the docked poses of melatonin with NS3Hel or NS5RdRp were generated in the grid-box dimension of 180 × 180 × 180 using the Lamarckian Genetic Algorithm. Other docking parameters followed the standard AutoDock protocol for ligand docking. The 1000 docked poses of melatonin and each JEV protein were generated and clustered by their Cartesian coordinates, with a 2 Å cluster radius. Then, the top 10 docked poses were ranked from all clusters by 2 criteria, i.e., the lowest interaction energy and the highest frequency of the docked poses. To investigate the possible binding of melatonin to JEV proteins, the hydrophobicity surface value of the selected top 10 docked poses was used to predict the interacting area of melatonin on each protein.

### Apoptosis and caspase activity assays

To examine the neuroprotective activity of melatonin against JEV-induced cell death, the apoptosis rate of JEV-infected cells was determined after treatment. SH-SY5Y cells were seeded in 24-well plates, and prophylactically treated with melatonin as described above. After 24 and 48 h of treatment, the infected cells were examined for apoptosis by the Muse Annexin V & Dead Cell kit (Cat#MCH100105, MilliporeSigma, Burlington, MA, USA). In addition, total caspase activity was measured using the Muse™ Caspase-3/7 Assay kit (Cat#MCH100108, MilliporeSigma). Both assays were performed according to the manufacturer’s protocol and were analyzed using a Muse^®^ Cell Analyzer (MilliporeSigma). The total apoptosis rate and caspase activity are presented as a percentage of the total cells.

### Quantitative real-time PCR (RT-qPCR)

SH-SY5Y cell monolayers in 6-well plates were pre-treated with or without melatonin for 1 h before JEV infection. JEV-infected cells were incubated with or without melatonin. Cells were harvested 24 and 48 hpi to evaluate the expression of apoptosis-associated genes, including BCL-2 associated X (*BAX),* B-cell lymphoma 2 *(BCL-2),* caspase-9 *(CAS9),* and caspase-3 *(CAS3),* and the expression of inflammation-related genes, including tumor necrosis factor-alpha (*TNF-α),* nuclear factor kappa B *(NF-κB),* cyclooxygenase-2 (*COX-2*) and Toll-like receptor (*TLR*) genes (*TLR3, TLR7*), in JEV-infected SH-SY5Y cells.

Total RNA was isolated from cells using TRIzol reagent (Invitrogen, Carlsbad, CA, USA), and each RNA sample was reverse-transcribed to complementary DNA (cDNA) by a SuperScript^®^ III First-Strand Synthesis System for RT-PCR (Invitrogen). The cDNA was used for SYBR-based real-time PCR analysis. The details of the primers used in this study and the optimal annealing temperatures are presented in Table 1. PCR amplification was performed using SsoAdvanced™ Universal SYBR Green Supermix (Bio-Rad Laboratories, Hercules, CA, USA). The reactions for the samples and no-template controls were run in duplicate. The amplification reaction and melt curve analysis were performed using a Bio-Rad CFX96 Thermal Cycler (Bio-Rad Laboratories), and the data were analyzed using CFX software (Bio-Rad Laboratories). *GAPDH* was selected as a housekeeping gene. The expression fold changes were calculated using the comparative Ct method. The expression level of each gene was expressed relative to the level in the uninfected control cells.

**Table 1:** The detail of primers used for qPCR.

Gene name	PrimePCR assay ID (Bio-Rad Laboratories)		Ta (°C)
*TNFα*	qHsaCED0037461		60
*NFκB*	qHsaCED00025636		60
	Primer sequence (5′-3′)		
*BAX*	Forward primer	CCCGAGAGGTCTTTTTCCGAG	60
	Reverse primer	CCAGCCCATGATGGTTCTGAT	
*BCL2*	Forward primer	TCGCCCTGTGGATGACTGA	60
	Reverse primer	CAGAGACAGCCAGGAGGAATCA	
*CAS3*	Forward primer	ACATGGCGTGTCATAAAATACC	60
	Reverse primer	CACAAAGCGACTGGATGAAC	
*CAS9*	Forward primer	CTCAGACCAGAGATTCGCAA	65
	Reverse primer	CTCAAGAGCACCGACATCAC	
*COX2*	Forward primer	CCCTTGGGTGTCAAAGGTAA	60
	Reverse primer	GCCCTCGCTTATGATCTGTC	
*TRL3*	Forward primer	TTGCCTTGTATCTACTTTTGGGG	60
	Reverse primer	TCAACACTGTTATGTTTGTGGGT	
*TRL7*	Forward primer	GATAACAATGTCACAGCCGTCC	56
	Reverse primer	GTTCCTGGAGTTTGTTGATGTTC	
*GAPDH*	Forward primer	CAGCCTCAAGATCATCAGCA	60
	Reverse primer	TGTGGTCATGACTCCTTCCA	

### Immunofluorescence assay

The viral envelope protein was detected by indirect immunofluorescence in JEV-infected cells. The SH-SY5Y cells were seeded in a cell culture slide chamber for microscopic fluorescence analysis for 24 h. Then, the cells were inoculated with JEV at an MOI of 0.1 for 1 h. After virus adsorption, infected cells were washed with phosphate-buffered saline (PBS) and fixed sequentially with 4% paraformaldehyde for 10 min on ice then removed fixative and followed by washing with PBS. The fixed cells were immediately permeabilized with 1% triton-X-100 and washed in PBS. Cells were incubated with an anti-flavivirus enveloped protein (4G2) antibody (MilliporeSigma, Burlington, MA, USA), followed by a secondary antibody, fluorescein isothiocyanate (FITC)-conjugated goat anti-mouse IgG (MilliporeSigma) and cell nuclei were counterstained with DAPI (4’,6-diamidino-2-phenylindole) (Sigma-Aldrich). The immunoreactivity was visualized and photographed using a Nikon ECLIPSE 80i fluorescence microscope (Nikon, Tokyo, Japan).

### Statistical analysis

All data and graphs were analyzed using GraphPad Prism (v6.0, GraphPad, San Diego, CA, USA). Data are expressed as the means ± SD from three independent experiments. The significance of differences between groups was evaluated using a one-way analysis of variance (ANOVA) followed by a multiple comparison test as appropriate. Data were considered statistically significant at a *p* value < 0.05.

## Results

### Cytotoxicity of melatonin on SH-SY5Y cells

In this study, SH-SY5Y cells were used as the representative neuronal target of JEV. Immunofluorescence staining of the viral envelope protein demonstrated the susceptibility of SH-SY5Y cells to JEV infection (Fig. 1A).

Before investigating the antiviral effects of melatonin, we first evaluated the possible cytotoxicity of melatonin on SH-SY5Y cells at 24 and 48 h post-treatment using the MTS assay, which measures the viability of the cells. Melatonin caused a weak dose-dependent reduction in cell viability even after exposure to melatonin for 48 h. Cell viability remained above 90% in the presence of 31.25 μM up to 500 μM of melatonin both at 24 h (Fig. 1C) and 48 h (Fig. 1D). The CC_50_ of melatonin for SH-SY5Y cells was 4018 μM for 24 h (Fig. 1E) and 2174 μM and 48 h (Fig. 1F). There was no change in cell morphology in 31.25–1000 μM melatonin-treated cells as compared to the untreated cells, this was consistent with the results of the cell viability test both 24 and 48 h. This result demonstrated the low cytotoxicity of melatonin on SH-SY5Y cells. Thus, doses of melatonin below CC_50_ values, including 125, 250 and 500 μM, that exhibited greater than 90% cell viability were selected for further studies on antiviral effects. Our selected doses were the same as those previously used in the in vitro studies on antiviral effects of melatonin against other virus types^25–31^.

### Direct virucidal effect of melatonin on JEV

**Figure 2:**
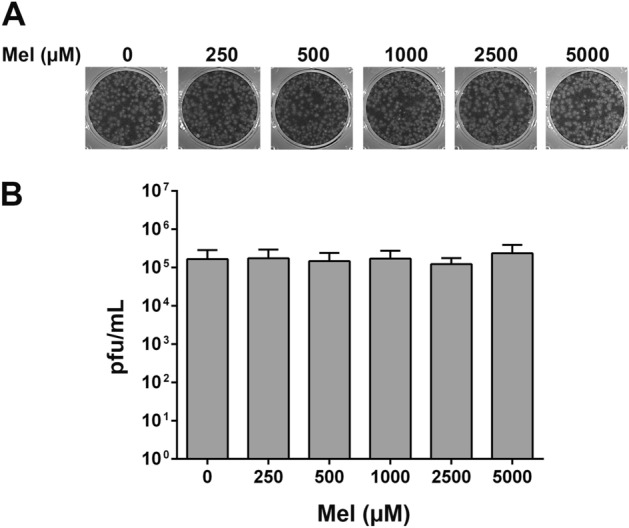
Analysis of the direct virucidal activity of melatonin (Mel) against Japanese encephalitis virus (JEV). (**A**) JEV was incubated with melatonin at various concentrations for 2 h, and the viral titer was determined by plaque assay and expressed in pfu/mL. (**B**) Representative plaque formation assay result. The graph shows the means ± SD of three independent experiments in technical triplicate. One-way ANOVA and Tukey–Kramer multiple comparisons tests were performed, and no statistical significance was detected.

To investigate whether melatonin directly eliminates the JEV particles, a virucidal activity assay was performed. JEV was incubated with melatonin at 250, 500, 1000, 2500 and 5000 μM for 2 h, and the remaining virus titers were evaluated by the plaque assay. The results showed no significant difference in virus titers at any dose of melatonin from the control without melatonin treatment (0 μM) (Fig. 2). The results suggest that melatonin is not virucidal to JEV particles.

### Melatonin reduces the release of infectious JEV progeny

**Figure 3:**
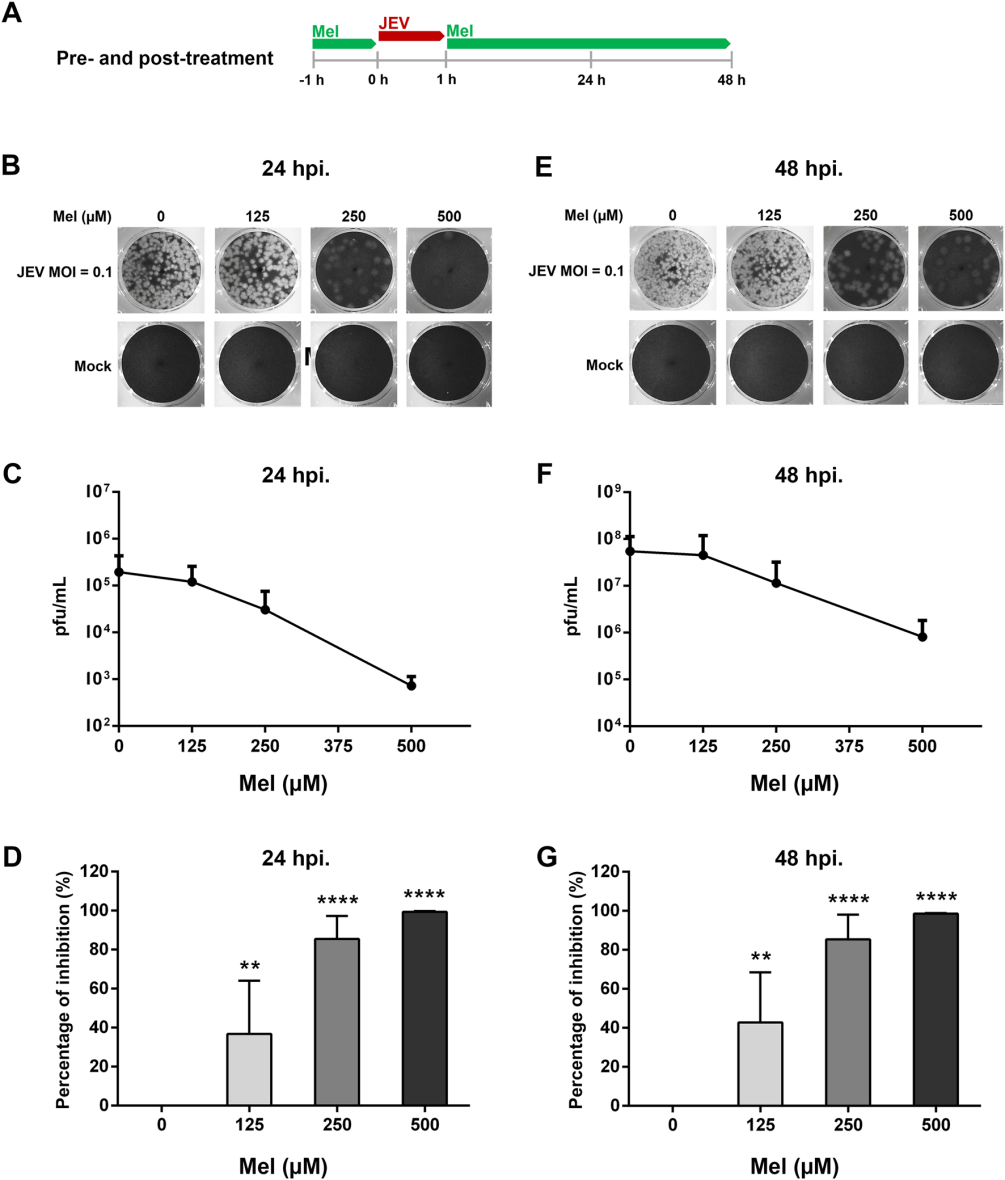
Antiviral effect of melatonin (Mel) on Japanese encephalitis virus (JEV)-infected SH-SY5Y cells in a pre- and post-infection treatment assay. (**A**) Schematic representation of the experimental design. The red line indicates the JEV absorption period and green line indicates the period when the cells were incubated with melatonin. SH-SY5Y cells were pre-treated with melatonin at various concentrations prior to infection with JEV at 0.1 MOI and then maintained in melatonin for 24 and 48 h. (**B**, **E**) Representative plaque formation assay results. Mock is uninfected cells treated with melatonin. (**C**, **F**) The viral titers in pfu/ml determined by plaque assay from cell culture supernatant, and (**D**, **G**) the reduction of viral titers expressed as percentage of inhibition. The graphs show the means ± SD of three independent experiments in technical triplicates. One-way ANOVA and Tukey–Kramer multiple comparisons tests were performed for statistical analysis. **p < 0.01 and ****p < 0.0001 compared to the control without melatonin treatment (0 μM).

As shown in previous reports of the protective effect of melatonin pre-treatment in various viral infection models, we first analyzed the prophylactic effect of melatonin by treating the cells before and after JEV infection (Fig. 3A). Melatonin treatment before and after JEV adsorption showed a significant dose-dependent decrease in viral yield in SH-SY5Y cells at both 24 hpi (Fig. 3B) and 48 hpi (Fig. 3E). Mock is uninfected cells treated with melatonin. Plaque forming assays demonstrated that melatonin treatment reduced the viral titer from 1.95 × 10^5^ to 7.25 × 10^2^ pfu/mL at 24 hpi (Fig. 3C) and from 5.47 × 10^7^ to 8.11 × 10^5^ pfu/mL at 48 hpi (Fig. 3F). A significant inhibitory effect of melatonin was observed for all tested concentrations at both time points (Fig. 3D and G). Up to a 99% inhibition or 2-log reduction in the virus yield was achieved at 500 μM melatonin at both 24 and 48 hpi (Fig. 3D). These results revealed that melatonin exhibited antiviral activity against JEV production in SH-SY5Y cells.

### Time-of-addition assay of melatonin against JEV

**Figure 4:**
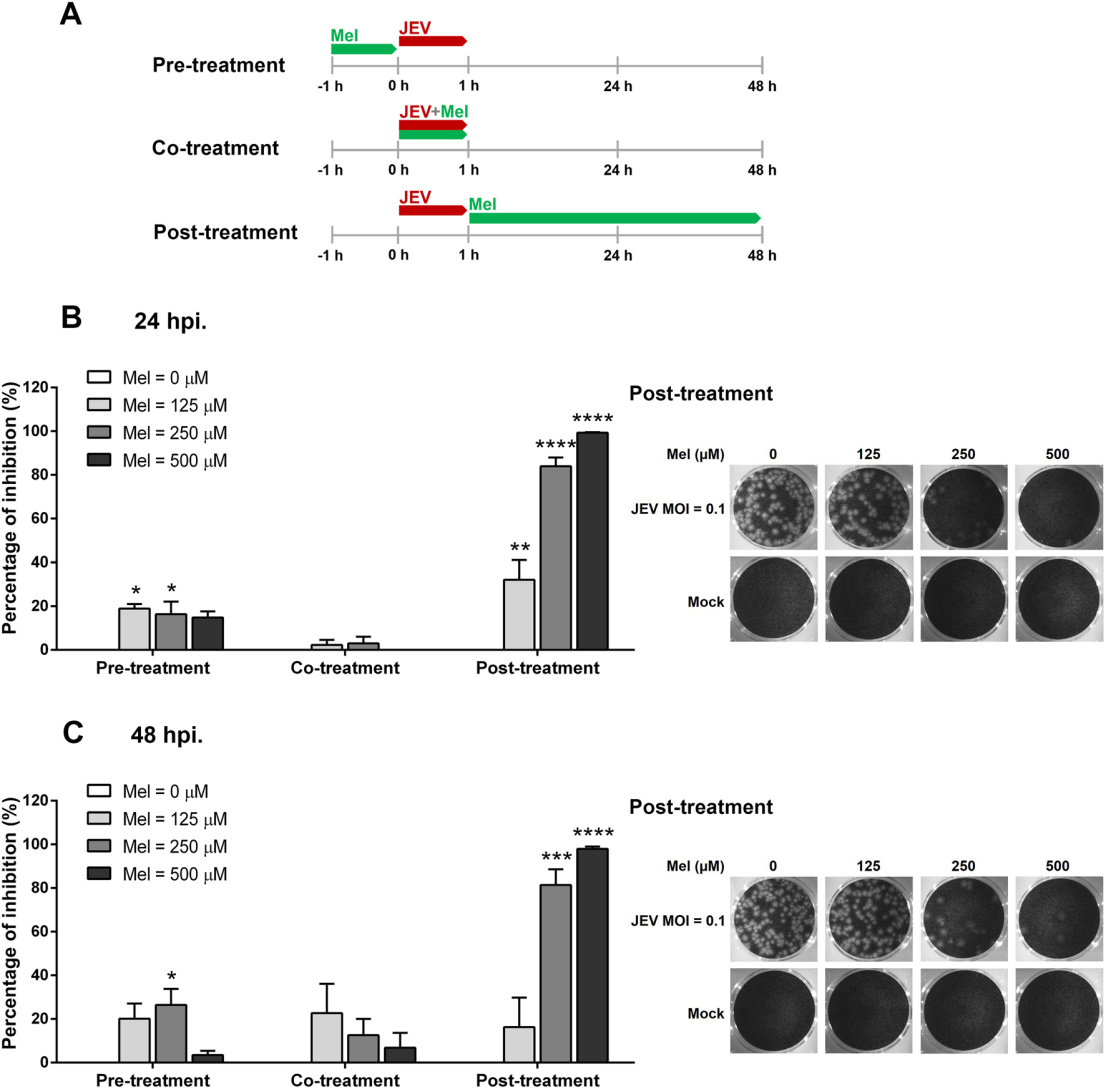
Time-of-addition experiments of melatonin (Mel) on Japanese encephalitis virus (JEV)-infected SH-SY5Y cells. (**A**) Schematic representation of the experimental design. The red line refers to the JEV absorption period and green line refers to the period when the cells were incubated with melatonin. Cells were infected with 0.1 MOI of JEV. Following melatonin treatment regimens were compared: (1) pre-treatment with melatonin for 60 min prior to JEV infection; (2) co-treatment with melatonin during JEV infection; (3) post-infection treatment with melatonin after JEV infection. Cells that were not treated with melatonin served as a control of virus infection. The viral titers were determined by plaque assay of cell culture supernatant and expressed as percentage inhibition at (**B**) 24 hpi and (**C**) 48 hpi. Representative plaque formation results from a post-infection treatment experiment is shown. The graphs show the means ± SD of three independent experiments in technical triplicates. One-way ANOVA and Tukey–Kramer multiple comparisons tests were performed for statistical analysis. *p < 0.05, **p < 0.01, ***p < 0.001 and ****p < 0.0001, compared to the control without melatonin treatment (0 μM).

We then identified the stages in which melatonin might be most effective in inhibiting JEV replication by performing time-of-addition assays. Various concentrations of melatonin were added as pre-treatment (prior to infection), co-treatment (at the same time as infection), and post-treatment (after virus entry) to JEV-infected cells (Fig. 4A). The percent inhibition was evaluated after 24 and 48 h of incubation and is shown in Fig. 4B (24 h) and 4C (48 h). Pre-treatment and co-treatment with melatonin showed slight or no effect on JEV titer levels at both 24 and 48 hpi, which suggests that melatonin may not affect the binding or entry of JEV into SH-SY5Y cells. In contrast, as demonstrated by plaque forming assays, post-treatment with melatonin significantly decreased JEV titers at 24 and 48 h of incubation. Melatonin inhibited JEV progeny release in a concentration-dependent manner in samples treated post-infection. A significant inhibitory effect of melatonin was observed at 125 μM (*p* < 0.01 at 24 hpi), 250 μM (*p* < 0.0001 at 24 hpi, and *p* < 0.001 at 48 hpi) and 500 μM (*p* < 0.0001 at 24 hpi, and *p* < 0.0001 at 48 hpi) when compared with untreated JEV-infected cells. At 500 μM melatonin post-treatment, a 2-log reduction of virus titer was observed at 24 and 48 hpi. The results demonstrated a potent inhibitory action of melatonin at the stage of viral replication.

### Molecular docking

**Figure 5:**
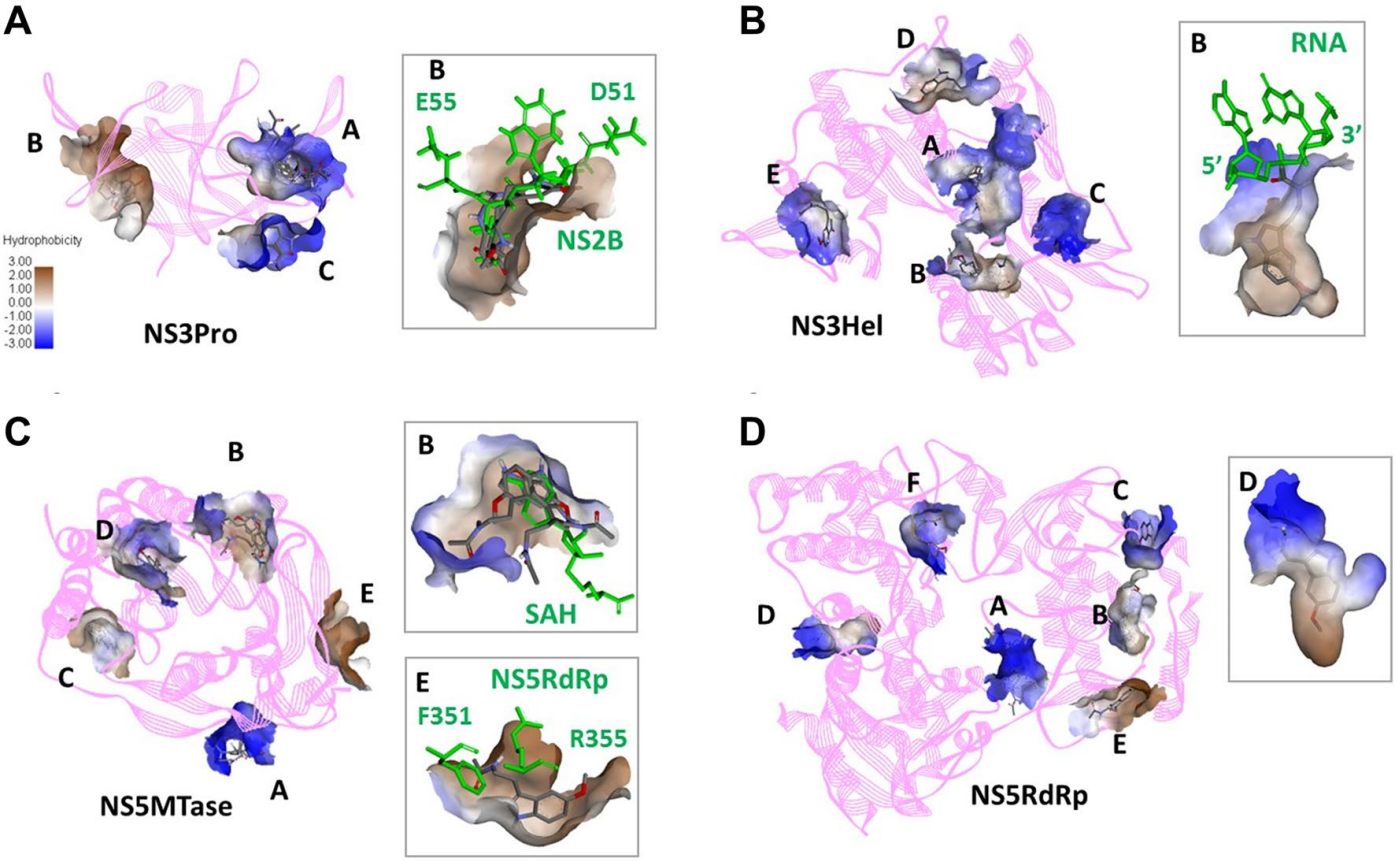
Molecular docking of melatonin with the JEV replicational enzymes NS3Pro, NS3Hel, NS5Hel, and NS5RdRp. The interaction areas of each enzyme with melatonin are indicated as regions A to F. The hydrophobicity value of the melatonin binding region reported in Table 2 is illustrated as a color spectrum. Gray indicates a positive hydrophobicity value, while blue indicates a negative hydrophobicity value. The right panels depict the superimposition of melatonin and the specific ligand with each enzyme. (**A**) The melatonin-docked NS3Pro superimposed on NS3Pro-NS2B (PDB ID: 4R8T). A detailed comparison of the docked melatonin ligands and NS2B is depicted in the zoomed view to the right. (**B**) The superposition of melatonin-docked NS3Hel with DENV RNA-NS3Hel (PDB ID: 2JLY^97^). An enlarged view of the docked melatonin and RNA (2GA3) is shown on the right. (**C**) The superimposition of NS5MTase with docked melatonin ligands and NS5MTase-SAH (PDB ID: 4K6M). Coordinate comparisons for melatonin ligands with SAH and NS5RdRp residues are explained in the right panels. (**D**) The docked melatonin on NS5RdRp. The images were produced using BIOVIA. (https://www.3ds.com/products-services/biovia/).

To gain a deeper mechanistic insight, we screened for possible intermolecular interactions between melatonin and JEV proteins that are vital for successful virus replication. The JEV replication enzymes, namely, NS3Pro, NS3Hel, NS5MTase and NS5RdRp, were evaluated with blind melatonin docking using a molecular docking approach. Then, the top 10 docked poses of each viral enzyme ranked by docking energy were further evaluated for the hydrophobic surface value to determine the interaction area with melatonin. As shown in Table 2, the binding region of the top 10 docked poses of each enzyme was categorized into several regions designated regions A to F. NS3Pro exhibited three different conformations in individual melatonin interactions. NS3Pro region B might be the most interesting interaction area since this region provided the lowest docking energy, ranging from − 6.07 to − 6.25 kcal/mol, with the highest positive hydrophobic surface value. Moreover, two docked poses, IDs 6 and 7, in region B were the top 3 highest number in cluster poses. Interestingly, the superimposed image of docked melatonin ligands in this hydrophobic region B overlapped with the binding region of NS3Pro and NS2B (Fig. 5A), where the two-component protease NS2B/NS3 promoted the processing of the viral polyprotein precursor to the mature viral protein. This might indicate that melatonin negatively interferes with JEV replication by interfering with the function of NS2B/NS3 protease. There were five melatonin binding grooves found in NS3Hel and NS5MTase, whereas NS5RdRp contained 6 different melatonin binding grooves. As shown in Fig. 5B, region B of NS3Hel seemed to be the likely interacting area. Although this region provided the lowest docking energies of − 6.67 and − 7.27 kcal/mol with zero and negative hydrophobic values, respectively, this pocket was located on domain II, which is associated with ssRNA substrate binding. This might influence viral genomic RNA replication. Hydrophobic binding areas in region B and E might relate to the inhibitory action of melatonin on NS5MTase. The superimposed figure (Fig. 5C) demonstrated that melatonin was able to perfectly fit into region B and region E, which could hinder NS5Mtase enzyme activity by preventing s-adenosylhomocysteine (SAH) binding and NS5RdRp binding, respectively. Even though the highest hydrophobicity value was found in region C of NS5MTase, this region was not associated with enzymatic function. As a consequence, melatonin may inhibit NS5MTase through two mechanisms: methylation of viral RNA and viral genome replication. In NS5RdRp, the last JEV enzyme we evaluated, one of six binding grooves, namely, region D, was considered crucial to NS5RdRp function since this region corresponds to the motif A of RdRp activity. Altogether, the computational approach revealed several possible intermolecular interactions of melatonin on JEV replication enzymes, NS3 and NS5, which might expedite the impairment of enzyme folding and catalytic function leading to the interference of JEV viral replication.

**Table 2:** The docked melatonin coordinates on JEV replication enzymes.

NS3Pro			NS3Hel			NS5MTase			NS5RdRp		
ID	LowE	HP	ID	LowE	HP	ID	LowE	HP	ID	LowE	HP
A											
1*	− 6.71	− 5.3	3	− 6.79	− 20.3	1*	− 6.91	− 18.1	1*	− 7.21	− 20.1
2	− 6.59	− 13.3	5*	− 6.66	− 8.4	5	− 6.24	− 21.9	2	− 7.15	− 19.7
3	− 6.58	− 6	7*	− 6.63	− 22.4	7	− 6.17	− 25.8	5	− 6.82	− 26.6
4	− 6.51	− 5.2	10	− 6.47	− 15.8	8	− 6.16	− 21.9	8	− 6.61	− 21.8
5	− 6.33	− 1.1									
9	− 6.11	− 10.7									
B											
6*	− 6.25	16.5	1	− 7.27	0	6	− 6.23	0.9	3	− 6.97	3.1
7*	− 6.23	13.7	6	− 6.64	− 2.9	9	− 6.16	− 1.9	4	− 6.95	− 3.9
10	− 6.07	18.3				10	− 6.15	− 0.3	6	− 6.73	− 5.3
C											
8	− 6.14	− 13.9	2	− 6.82	− 27	2	− 6.54	14.2	7	− 6.71	− 14.1
			4	− 6.72	− 22.2	3	− 6.43	17.8			
D											
			8	− 6.56	− 6.2	4	− 6.25	− 16	10	− 6.59	3.8
						24*	− 5.93	− 2.8			
E											
			9*	− 6.55	− 7.8	27*	− 5.86	11.7	20*	− 6.14	17.4
F											
									22*	− 6.14	− 18.2

*Note* ID, Nomenclature for docking poses; LowE, Docking energy (kcal/mol); HP, Hydrophobicity of groove interacting to an individual docked melatonin; the asterisk above ID names refers to the top 3 highest-number-in-cluster poses.

### Effect of melatonin on JEV-induced SH-SY5Y cell apoptosis

**Figure 6:**
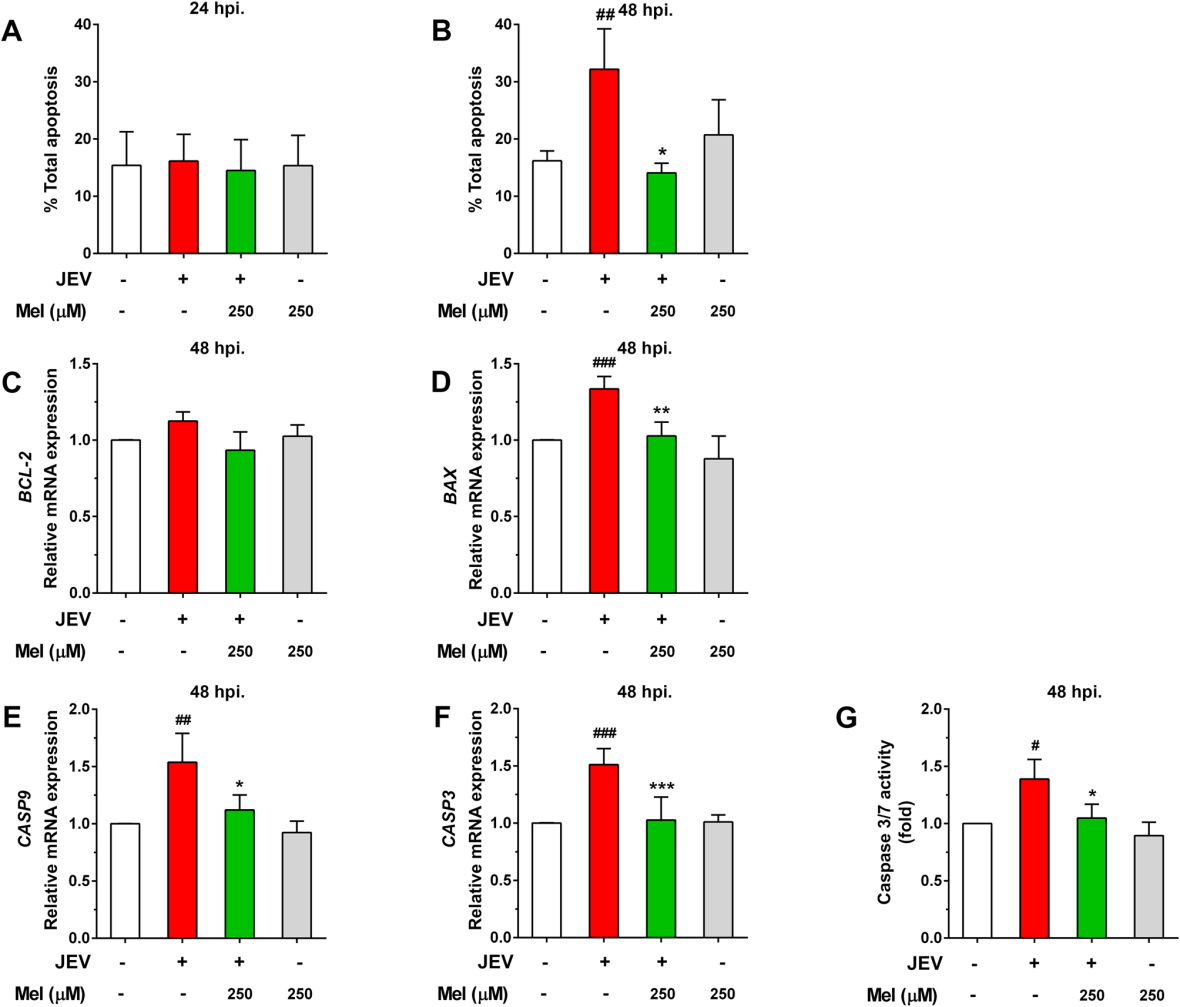
The effect of melatonin (Mel) on the apoptosis pathway in JEV-infected SH-SY5Y cells. Cells were pre-treated with melatonin at various concentrations and infected with 0.1 MOI of JEV. The cells were maintained in the presence of melatonin for 24 or 48 h. The infected cells were collected, and the number of apoptotic cells was measured by flow cytometry at (**A**) 24 hpi and (**B**) 48 hpi. The mRNA expression of apoptosis-related genes at 48 hpi was determined by qPCR. (**C**) *BCL-2*, (**D**) *BAX*, (**E**) *CAS9*, and (**F**) *CAS3*. (**G**) The caspase-3/7 activity of JEV-infected cells was measured at 48 hpi. The graphs show the means ± SD of three independent experiments. One-way ANOVA and Tukey–Kramer multiple comparisons tests were performed for statistical analysis. #*p* < 0.05, ##*p* < 0.01, and ###*p* < 0.001 compared to the cell control. *p < 0.05, **p < 0.01, and ***p < 0.001 compared with the JEV-infected cells.

The effect of melatonin on the induction of neuronal cell death by JEV infection and the expression of cell signaling molecules in apoptosis pathways were investigated; the results are presented in Fig. 6. The dose of melatonin at 250 μM was selected for the experiment. The protective effect of melatonin on neuronal cell apoptosis during JEV infection was evaluated by measuring the apoptosis rate. Cells were stained with annexin V and 7-AAD, and analyzed by flow cytometry. Our results showed that 250 μM of melatonin did not influence the apoptosis induction of SH-SY5Y cells compared to control cells at either 24 h (Fig. 6A) and 48 h (Fig. 6B). During the first 24 hpi, there was no significant change in the percentage of apoptotic cells in JEV-infected cells regardless of melatonin treatment compared to uninfected control cells (Fig. 6A). However, at 48 hpi, infection with JEV induced a significant number of apoptotic SH-SY5Y cells when compared to uninfected control cells (*p* < 0.01, Fig. 6B), which was consistent with our previous findings of the apoptosis kinetics in SH-SY5Y cells after JEV infection^5^. Melatonin treatment reduced the SH-SY5Y cell apoptosis induced by JEV infection (*p* < 0.05, Fig. 6B).

We further investigated whether melatonin protected against JEV-triggered neuronal death via the intrinsic apoptosis pathway. JEV infection showed no significant change in the percentage of apoptotic cells at the early 24 h of infection. Thus, the mRNA expression levels of genes involved in the apoptosis pathway were measured only at 48 hpi. The results showed that the mRNA levels of *BAX* (*p* < 0.001, Fig. 6D)*, CAS9* (*p* < 0.01, Fig. 6E)*,* and *CAS3* (*p* < 0.001, Fig. 6F) were significantly upregulated in the JEV-infected cells compared with the uninfected cells. Melatonin treatment significantly suppressed the increase in the mRNA levels of these apoptosis-related genes in JEV-infected cells (Fig. 6D–F). Moreover, we observed an increase in caspase-3/7 activity in JEV-infected cells (*p* < 0.05, Fig. 6G), which was consistent with elevated apoptotic gene expression and apoptotic cell death. Treatment with melatonin significantly reduced caspase-3/7 activity in JEV-infected cells (*p* < 0.05, Fig. 6G). These results demonstrate the anti-apoptotic effect of melatonin against neuronal death by JEV infection.

### Effect of melatonin on JEV-induced inflammation in neuronal cells

**Figure 7:**
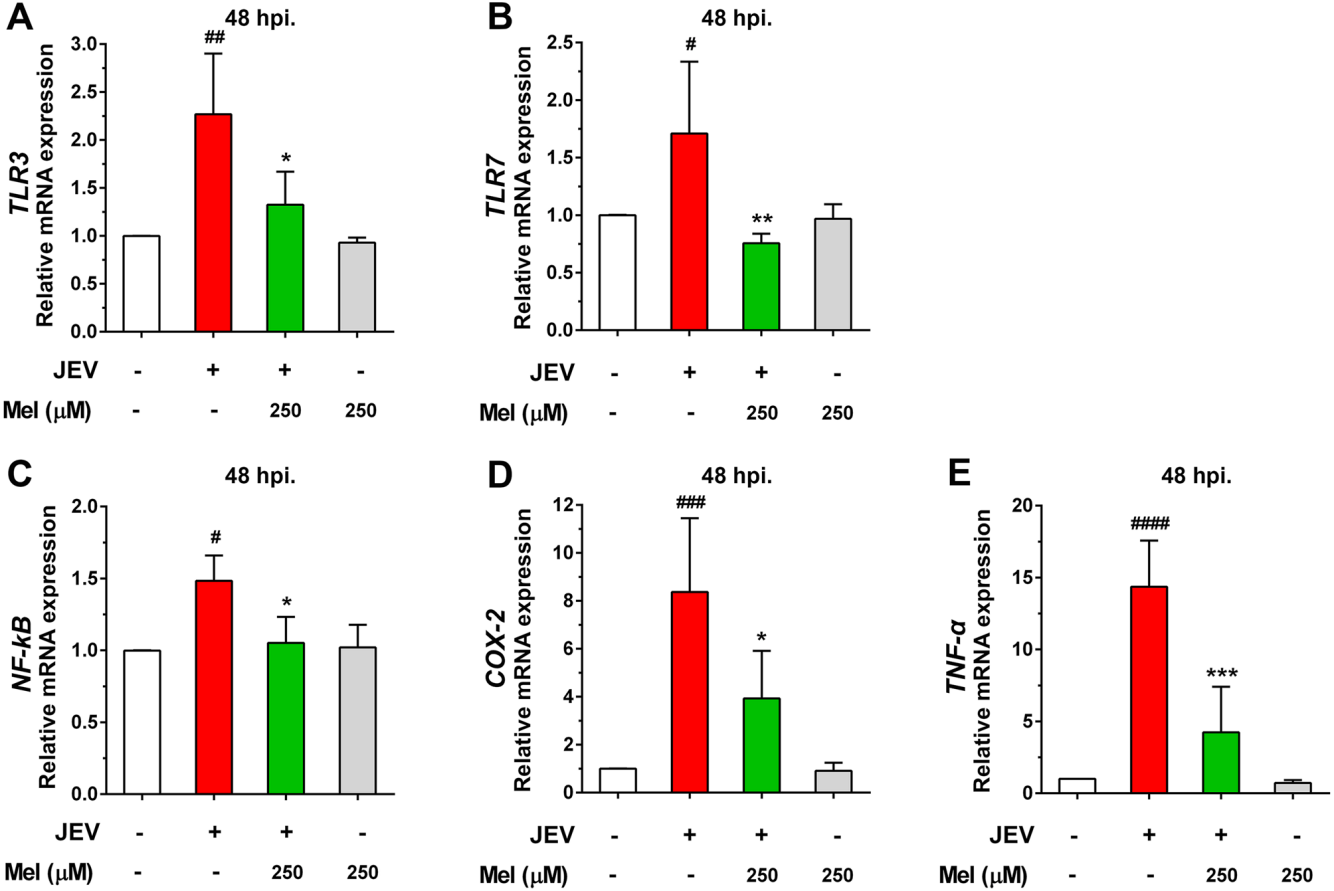
The effect of melatonin (Mel) on inflammation-related genes in JEV-infected SH-SY5Y cells. Cells were pre-treated with melatonin at various concentrations and infected with 0.1 MOI of JEV. The cells were maintained in melatonin for 24 or 48 h. The infected cells were collected to determine mRNA expression by qPCR. (**A**) *TLR3*, (**B**) *TLR7*, (**C**) *NF-κB*, (**D**) *COX-2*, and (**E**) *TNF-α.* The graphs show the means ± SD of three independent experiments. One-way ANOVA and Tukey–Kramer multiple comparisons tests were performed for statistical analysis. #*p* < 0.05, ##*p* < 0.01, ###*p* < 0.001, and ###*p* < 0.0001 compared to the cell control. *p < 0.05, **p < 0.01, and ***p < 0.001 compared with the JEV-infected cells.

To determine the effect of melatonin on the JEV-induced neuroinflammation, the expression of inflammation-related genes that can directly mediate neuronal apoptosis was evaluated. Previous research had shown that JEV induces inflammation by triggering the TLR signaling pathway^32,33^. TLRs such as TLR3 and TLR7 play critical roles in the recognition of viral nucleic acid components^34^. At 48 hpi, we found that JEV infection significantly increased the mRNA levels of the *TLR3* (*p* < 0.01, Fig. 7A) and *TLR7* (*p* < 0.05, Fig. 7B) genes in JEV-infected SH-SY5Y cells. JEV induced increases in the expression of the relevant inflammatory mediators *NF-κB* (*p* < 0.05, Fig. 7C) and *COX-2* (*p* < 0.001, Fig. 7D) and the proinflammatory cytokine *TNF-α* (*p* < 0.0001, Fig. 7E) at the mRNA level. Treatment with melatonin at 250 μM before and after infection significantly reversed the induction of *TLR*3 (*p* < 0.05) and *TLR*7 (*p* < 0.01) mRNA levels and suppressed the expression of *NF-κB* (*p* < 0.05)*, COX-2* (*p* < 0.05) and *TNF-α* (*p* < 0.001) induced by JEV infection (Fig. 7). Thus, melatonin might potentially ameliorate the severity of JEV-induced neuroinflammation by reducing the amplitude of TLR-mediated signaling molecule induction which results in lowering the production of inflammatory factors.

## Discussion

This study is the first to report on the antiviral efficacy of melatonin against JEV. We first evaluated the direct anti-JEV activity of melatonin by incubating the virus with melatonin; the results showed that melatonin had no virucidal effect as measured by the infectivity of JEV particles as shown for other viruses. We further evaluated the anti-JEV effect of melatonin in SH-SY5Y cells. Melatonin had anti-JEV activity, as evidenced by the time- and dose-dependent decrease in viral yield in the pre- and post-infection treatment assay. The time-of-addition assay indicated that melatonin exerted the major inhibitory activity on the post-entry step of the JEV replication cycle; this was demonstrated by the significant reduction of JEV yield when the cells were treated after infection, but not before or during infection.

In this study, the concentration of melatonin was chosen based on our cytotoxicity assessment and previous in vitro studies of the antiviral effect of melatonin against several viruses; these values were between 0.1 and 1.2 mM in different types of cell lines^25–31^. Previous studies have demonstrated the protective effect of melatonin at 0.1–1 mM against toxic substance-induced cytotoxicity, oxidative stress, and apoptosis in neuronal and other cells^35–38^. Numerous earlier studies have documented the high safety profile of melatonin; a high dose greater than 200 mg/kg of melatonin had no adverse effects in in vivo experiments^39–42^. Clinical use of melatonin also shows no substantial adverse effects when melatonin was administered for long-term treatment^43,44^.

Previously, melatonin supplementation was suggested to be effective against several viral infections both in vitro and in vivo^18,45,46^. Treatment with melatonin lowered the level of virus in blood and in target cells and increased the survival rate of animals infected with Venezuelan equine encephalomyelitis virus (VEEV)^47^, Semliki Forest virus (SFV)^48^, WNV-25^48^, influenza A virus^25^, rabbit hemorrhagic disease virus (RHDV)^49,50^, ZIKV^30^ and swine coronaviruses^31^. Currently, the use of melatonin as a treatment in patients with SARS-CoV-2 infection is under clinical trials^51–54^. The present in vitro results demonstrated that 125 to 500 μM melatonin significantly reduced the viral titer 48 h after JEV infection. The robust inhibitory effect of melatonin in the JEV post-entry stage could indicate that melatonin may exert antiviral effects against JEV by interfering with the replication cycle of JEV. These results will serve as the basis for establishing the effective dosage and timing of melatonin administration for treating JEV infection. In addition, our computational analysis of the predicted interaction of melatonin with JEV proteins that function in the post replication processes revealed the possible mechanism of melatonin-medicated inhibition of JEV production.

Melatonin reportedly binds to several enzymes such as quinone reductase 2 (QR2), notum, pepsin, and metalloproteinase-9 (MMP-9) in the hydrophobic cavity^55^. According to the crystallization data, the hydrophobic melatonin-binding pocket is located either in the enzyme active site, such as in QR2^56^, or near the substrate entrance to catalytic sites, such as in notum^57^. The docking approach revealed similar results: melatonin binds to the catalytic domain of MMP-9^58^ or to the non-enzymatic site in pepsin and changes its physiological function^59^. Several studies have proposed that melatonin impacts viral replication; however, the mechanisms of melatonin action on JEV viral proteins are not well understood. Here, we described our findings on the melatonin-mediated interference of JEV replication by focusing on the hydrophobic groove in the melatonin-binding JEV enzymes. The hydrophobic groove is important for their enzymatic activities or allosteric regulations. The non-structural proteins NS3 and NS5 are attractive targets for evaluating the role of melatonin because these two enzymes are key components of the replication-competent complex. The NS3 protein, which plays pivotal functions in JEV viral protein maturation and RNA replication, consists of an N-terminal protease domain (NS3Pro), which requires NS2B for its activity, and a C-terminal RNA helicase domain (NS3Hel). NS5 carries an N-terminal RNA methyltransferase (NS5MTase) domain and a C-terminal RNA-dependent RNA polymerase (NS5RdRp) domain, which is a key component of the viral RNA complex in the JEV replication process^60^. The four separate structures, including NS3Pro, NS3Hel, NS5MTase and NS5RdRp, have been characterized by crystal structures with and without their inhibitors and ligands. In this study, the docking analysis indicated that all of these proteins are possible targets of melatonin binding, which might account for the reduction of JEV production.

The docking simulation investigated whether melatonin negatively affected viral replication by interfering with physiological function and/or enzymatic activity. Highly hydrophobic melatonin-binding surfaces were found in NS3Pro (region B) and NS5MTase (region E), which are important for enzymatically active conformation, while mild hydrophobicity was associated with catalytic function in NS3Hel (region B), NS5MTase (region B), and NS5RdRp (region D), as shown in Table 2 and Fig. 5. As for the effect of melatonin on enzyme folding in the active state, melatonin might adversely affect NS3Pro by interfering with the interaction between the hydrophobic groove of N-terminal NS3Pro and N-terminal NS2B residues 51–55 (Fig. 5A). This N-terminal NS2B is confirmed to be sufficient to stabilize NS3Pro in both catalytically inactive and active states^61^, and the mutagenesis analysis suggested that deletion of NS2B residues 51 to 55 abolished cleavage efficiency^62^. Additionally, as melatonin was able to dock into the hydrophobic surface of NS5MTase, which is an NS5RdRp-interacting module (Fig. 5C), melatonin would probably disrupt the correct rearrangement of the active platform for NS5RdRp, leading to physiological impairment of the MTase-RdRp interface. This MTase-RdRp hydrophobic network is essential for NS5RdRp movement^22,63^ and RdRp polymerase function in both the initiation of RNA synthesis and the elongation of RNA^64,65^.

To gain insight into the effect of melatonin on the enzymatic area in NS3Hel, docked melatonin was positioned on domain II, which is involved in ssRNA recognition. Through this mechanism, melatonin might impair the stable interaction between ssRNA and the unwinding channel by interfering with hydrogen bonding and/or hydrophobic forces between the two molecules (Fig. 5B). For NS5MTase, the overlay between docked melatonin and superimposed s-adenosylhomocysteine (SAH), the byproduct of methylation, at the S-adenosyl-L-methionine (SAM) binding pocket (Fig. 5C) suggested that this enzymatic motif could not accept a methyl group from a SAM methyl donor. Moreover, mutagenesis studies revealed that mutations in the SAM binding pocket abolished the methylation process^66,67^. In NS5RdRp, melatonin might negatively affect RdRp function by binding to the palm domain motif A, which is essential for RdRp catalysis^68^. In this study, molecular docking analysis revealed that melatonin might interfere enzymatic activities of NS3 and NS5 which lead to the inhibition of JEV replication. In order to investigate mechanistic insights of melatonin into the impairment of JEV NS3 or NS5 enzymatic functions, structure and functionality analysis using computational and experimental approaches need to be further studied to describe the molecular mechanisms of melatonin against NS3 and NS5 enzymatic functions. This could demonstrate the application of melatonin as a therapeutic agent to combat JEV infection in the future.

Due to its amphiphilicity, melatonin readily passes through the cell membrane while also entering cells via specific transporters^69–71^ where it influences intracellular physiology^11^. Additionally, melatonin transmits its actions via the two specific high-affinity G protein-coupled receptors that are present on the limiting membrane of cells and also on mitochondrial membranes^12,13,72^. Melatonin itself and its metabolites exert potent antioxidant and anti-inflammatory effects^73–75^. The intracellular actions of melatonin, especially its neuroprotective effect, have been documented in numerous models of neurological disease^76^. Melatonin also protects neuronal cells from numerous cytotoxic substances including several pathogens which normally induce neural damage^77,78^.

JEV directly targets neuronal cells in the CNS where it increases destructive cellular oxidative stress and induces neuronal apoptosis^5,6^. A JEV infection increases free radical and lipid peroxidation production while impairing antioxidant defense mechanisms^7^. The upregulation of oxidative stress by JEV infection also activated mitochondrial and endoplasmic reticulum stress, which resulted in the induction of apoptotic protein markers in the intrinsic apoptosis pathway^54,79,80^. Our previous report also showed that JEV infection elevated the production of highly deleterious apoptogenic molecules that often induce neuronal death, especially in the late stage of infection^5^. In the current study, melatonin treatment significantly attenuated JEV-induced apoptotic cell death, as evidenced by the reduced apoptosis rate 48 h after JEV infection. Moreover, melatonin ameliorated SH-SY5Y cell apoptosis caused by JEV infection by acting on the intrinsic apoptosis signaling pathway. Melatonin decreased the expression levels of proapoptotic molecules and subsequently reduced the activation of caspase in JEV-infected cells; this was accompanied by a reduction total number of apoptotic neurons induced by JEV infection. In line with several previous reports, the present results support the anti-apoptotic activity of melatonin in protecting neuronal cells from viral infection-induced death. Melatonin reduces oxidative damage by directly scavenging reactive oxygen species and nitric oxide, while it enhances indirect antioxidant potential by increasing antioxidant enzyme activities including glutathione peroxidase. As a result, melatonin reduces oxidative stress in virus-infected cells as presented in VEEV infection^26,29,50^, respiratory syncytial virus (RSV)^81^, coxsackievirus B3 (CVB3)^82^, and RHDV^50^. Melatonin also aids in protecting against virus-induced apoptotic death by regulating the antiapoptotic and proapoptotic signaling pathways in fighting viral infections^18,26,50,82^. Therefore, melatonin exhibits anti-apoptotic effects that protect against virus-induced apoptotic cell death through the regulation of the intrinsic apoptosis pathway, that is, by increasing anti-apoptotic proteins while lowering pro-apoptotic proteins and caspase cascade activity.

Inflammation is an important feature of a JEV infection. Previous reports have shown that JEV infection induces the migration of various immune cell types to the infected sites which promote the production of several neuroinflammatory cytokines that cause chronic inflammation and accelerate the progression of neuronal damage^8,83^. In this study, we reported increased expression levels of inflammation-related genes in SH-SY5Y cells after JEV infection. The upregulation of TLR signaling molecules and the increase in the levels of proinflammatory cytokines, such as *TNF-α,* in JEV-infected cells are consistent with previous reports^83–85^. Overproduction of TNF-α by JEV infection is one of the major causes of neuronal apoptosis via the TNF receptor-associated death domain^80^ which triggers inflammatory cells to release other cytokines, resulting in neuronal cell death^85,86^.

Multiple reports document melatonin as an anti-inflammatory agent during exacerbated immune responses, such as acute inflammation^77,87^. In this study, melatonin treatment attenuated JEV-induced overexpression of TLR-mediated signaling molecules in SH-SY5Y cells, which was associated with an increase in the survival rate of infected cells. As demonstrated for other viruses, melatonin exhibits anti-inflammatory effects both in vitro and in vivo by preventing the overproduction of proinflammatory cytokines such as TNF-α and a secondary surge of proinflammatory cytokines including interleukin-1, interleukin-6 and NF-κB in VEEV^88^, CVB3^82^, influenza A virus^25^ and RHDV^49^ infections. These results confirm the anti-inflammatory effects of melatonin, which shows that this molecule suppresses the excessive inflammatory response resulting from viral infections to prevent the adverse effects of proinflammatory cytokines on host cells.

**Figure 8:**
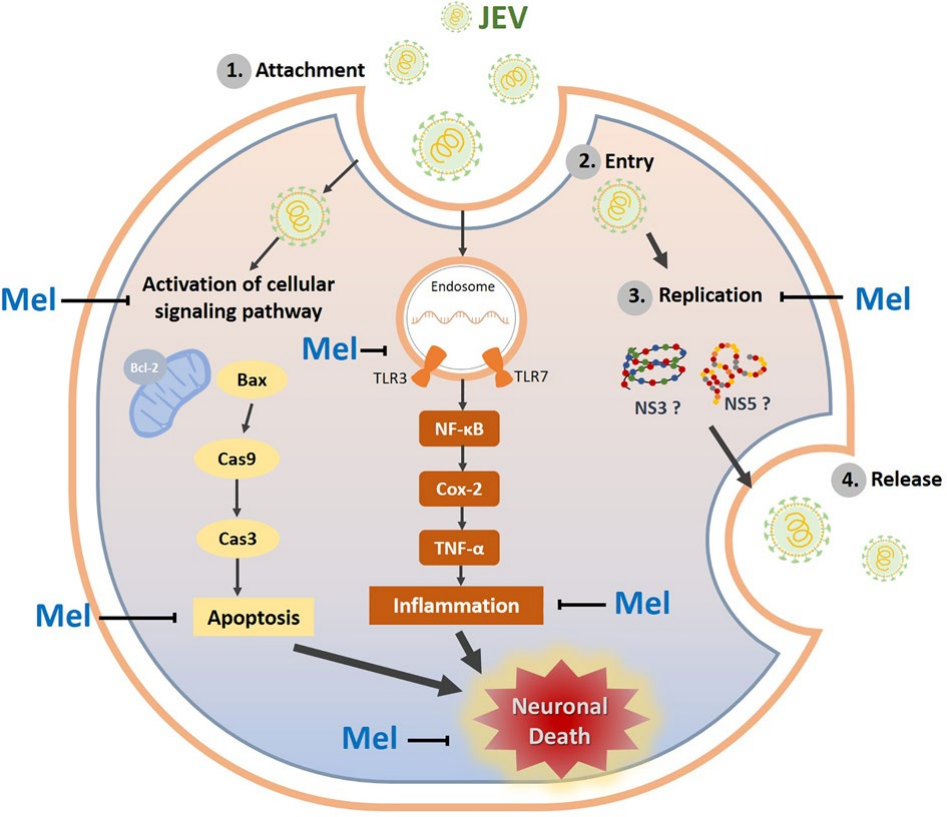
Schematic illustration of the proposed neuroprotective mechanisms of melatonin in preventing JEV-induced neuronal death. During infection of neuronal cells, (1) JEV virions attach to the cell, (2) virus enters the cell, (3) viral genome undergoes replication, transcription and translation and virions are assembled, and ultimately (4) virus is released. Melatonin (Mel) inhibits the production of new viral progeny by interfering with the replication cycle of JEV at the post-entry stage. Melatonin may exert this effect by abrogating the enzymatic function of NS3 and NS5 through binding to the active site or steric hindrance of enzymes. Additionally, melatonin attenuates the JEV-induced upregulation of TLRs, NF-κB and COX-2 signaling molecules, moderates the overproduction of TNF-α and prevents the adverse effects of inflammation on host cells. Moreover, melatonin prevents JEV-induced apoptotic cell death via the regulation of the intrinsic apoptosis pathway by reducing the levels of pro-apoptotic proteins and the activity of the caspase cascade. Melatonin functions as an antiviral agent and modulates host cell signaling pathways related to inflammation and apoptosis to protect against neuronal death induced by JEV infection. The schematic was produced using Microsoft PowerPoint.

In this study, we demonstrated the beneficial effects of pre- and posttreatment or prophylactic treatment with melatonin on JEV infection. We observed that melatonin functioned in multiple aspects of JEV infection including the interference of new viral progeny production and the neuroprotection of host cells through the inhibition of apoptosis and inflammation pathways (Fig. 8). As shown in earlier reports, pre-treatment or prophylactic treatment with melatonin confers neuroprotection in many neurological disease models^78,89–91^ as well as having therapeutic potential against many pathogens, such as parasites, bacteria and viruses^15,92,93^. Notably, the efficacy of melatonin as a prophylactic treatment for the prevention of symptomatic SARS-CoV-2 infection or for the suppression of the symptoms of the disease in SARS-CoV-2 infective individuals has been evaluated in clinical trials and shown to be effective in reducing the severity of the disease as well as reducing mortality^52,94–96^. Our present results provide evidence for the promising broad therapeutic and preventive efficacies of melatonin and suggest that melatonin could be further investigated for its diverse therapeutic actions for viral diseases.

## Conclusion

The present results identify the beneficial effect of melatonin as a potential molecule for the further development of antiviral agent against JEV. Melatonin may exert its function against JEV infection by suppressing viral progeny production and modulating various molecular targets in apoptosis and inflammation signaling pathways of the host (Fig. 8). The ability of melatonin to protect against JEV infections may have application for the treatment of Japanese encephalitis by delaying the pathogenesis in the CNS and relieving symptoms, which could enhance the survival rate, reduce the severity neurological sequelae and improve the quality of life of the patients.

## Data Availability

The data used to support the findings of this study are available from the corresponding author upon request.
